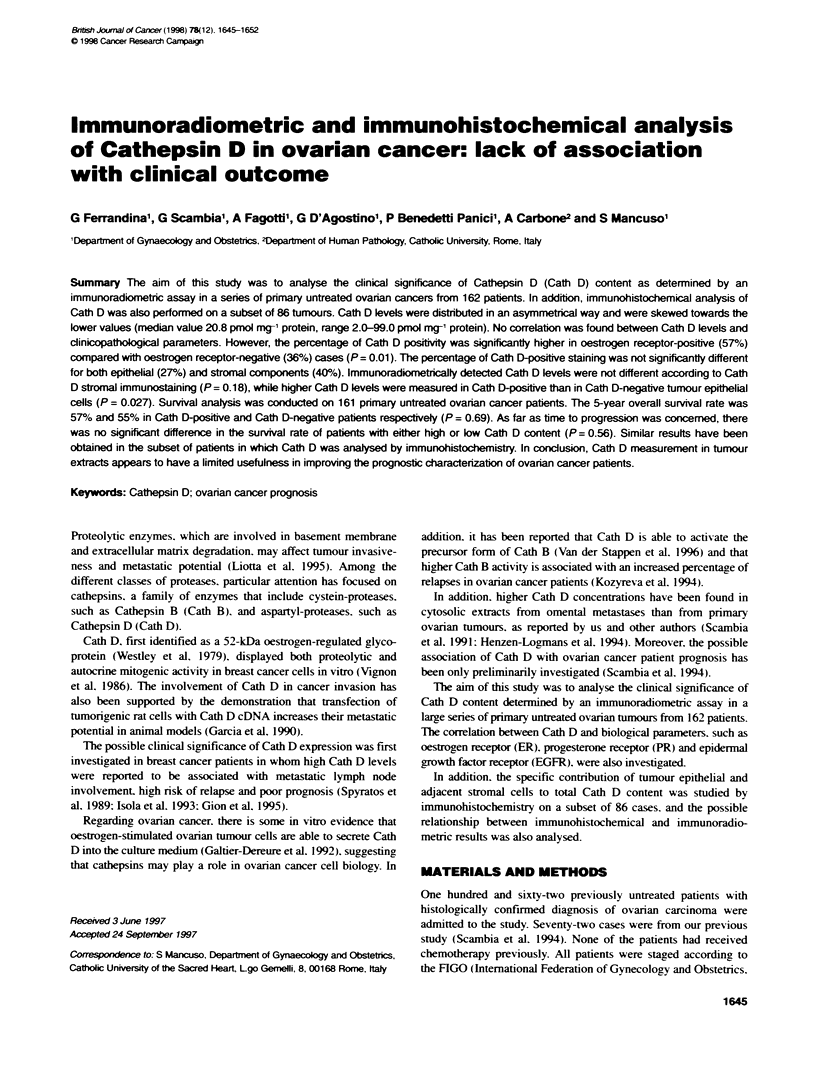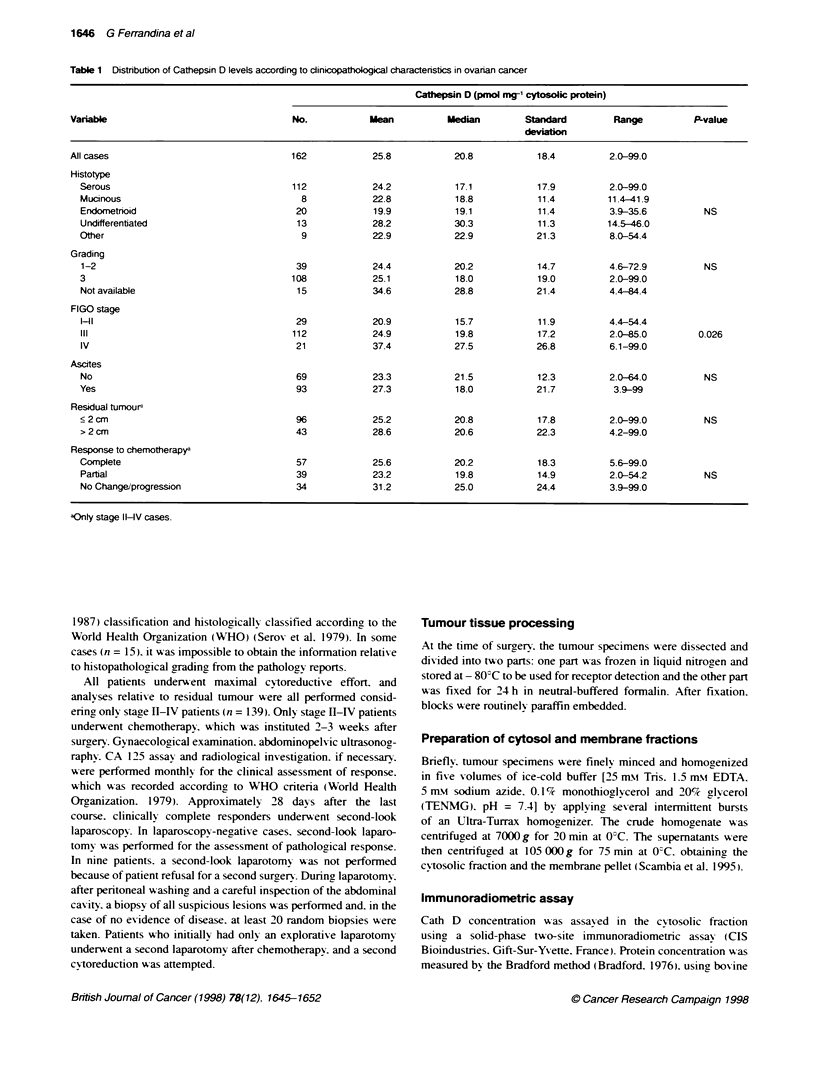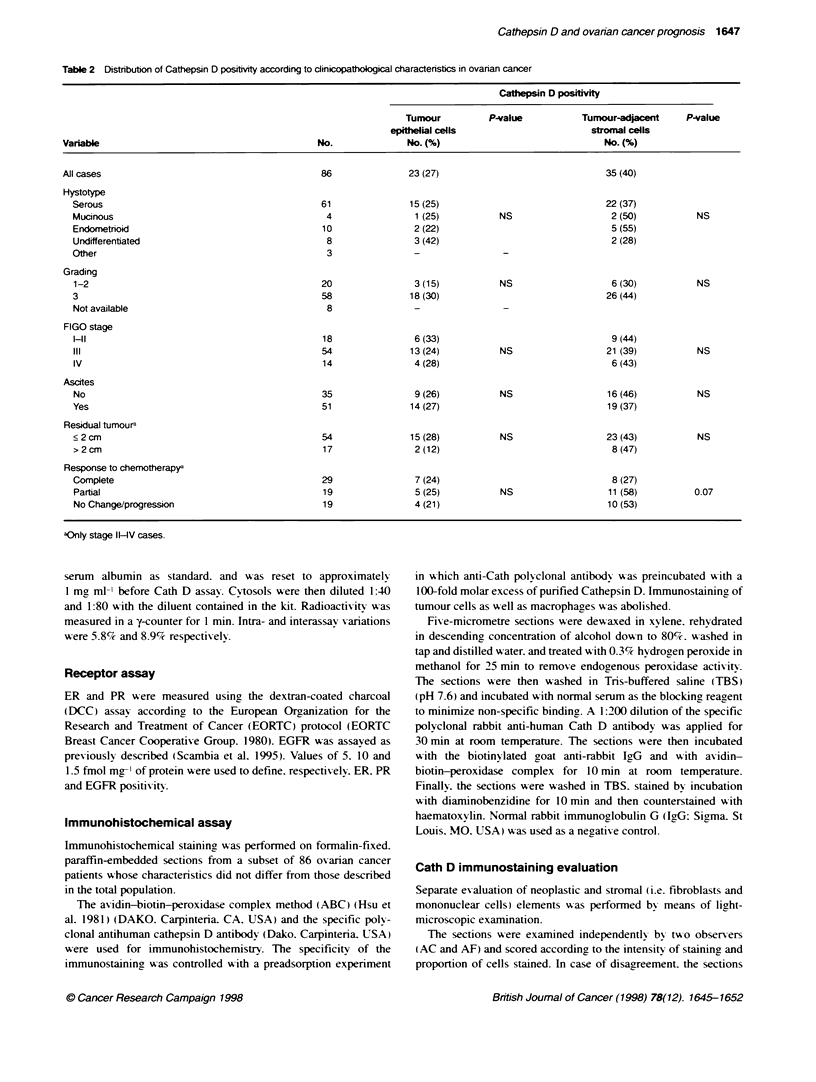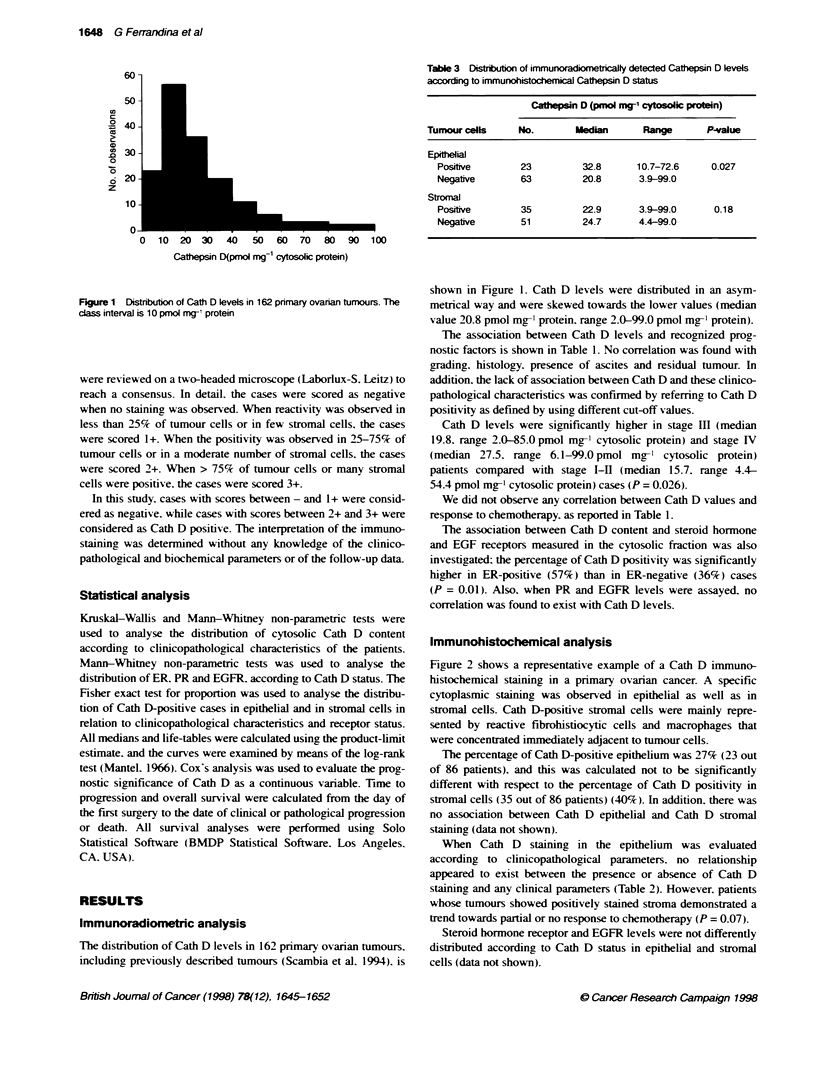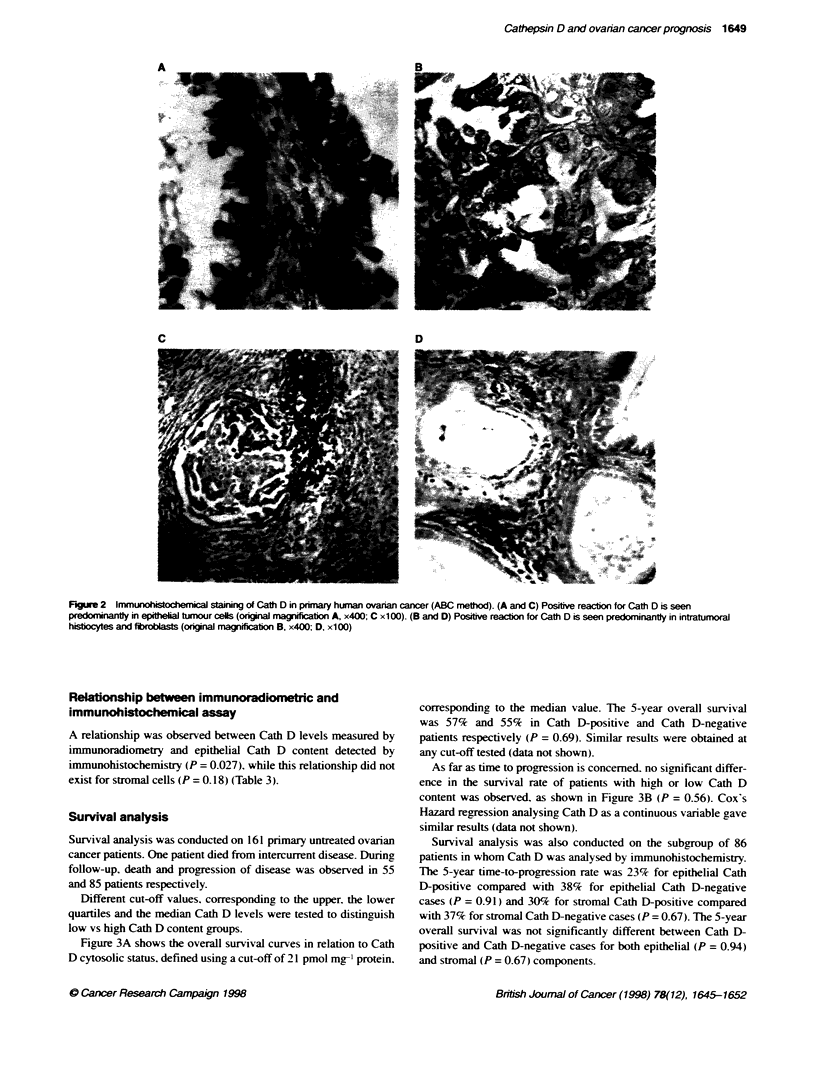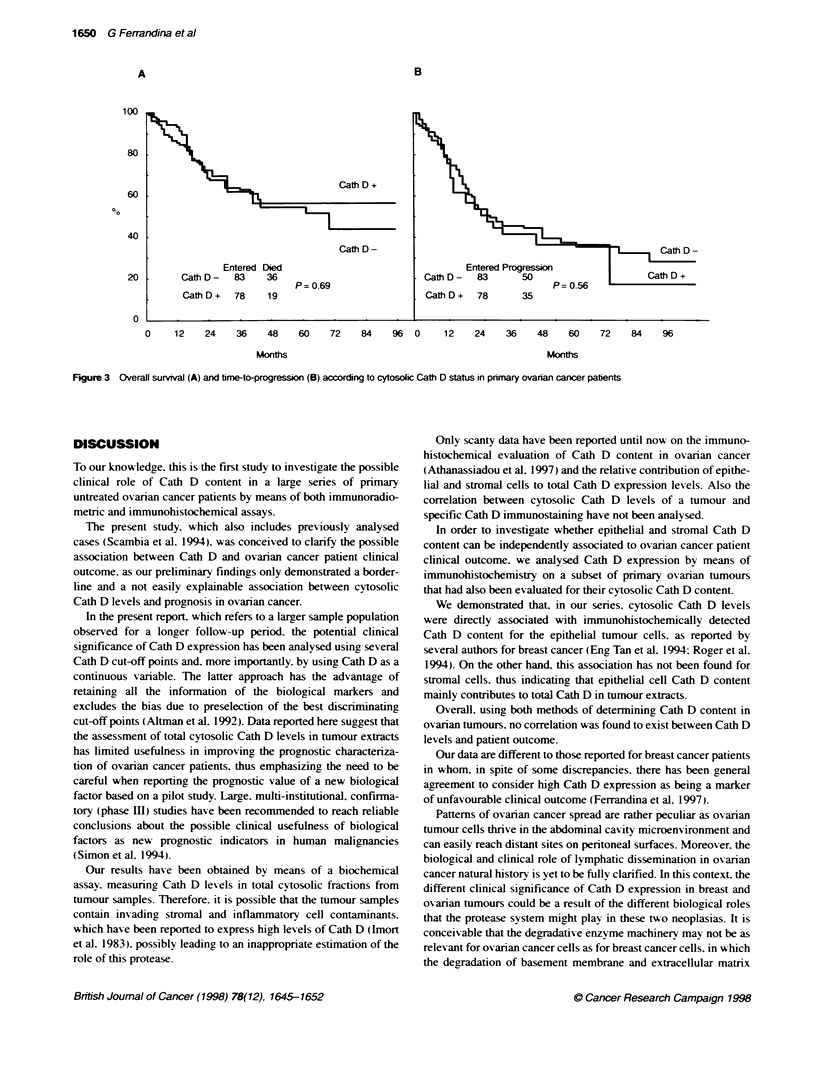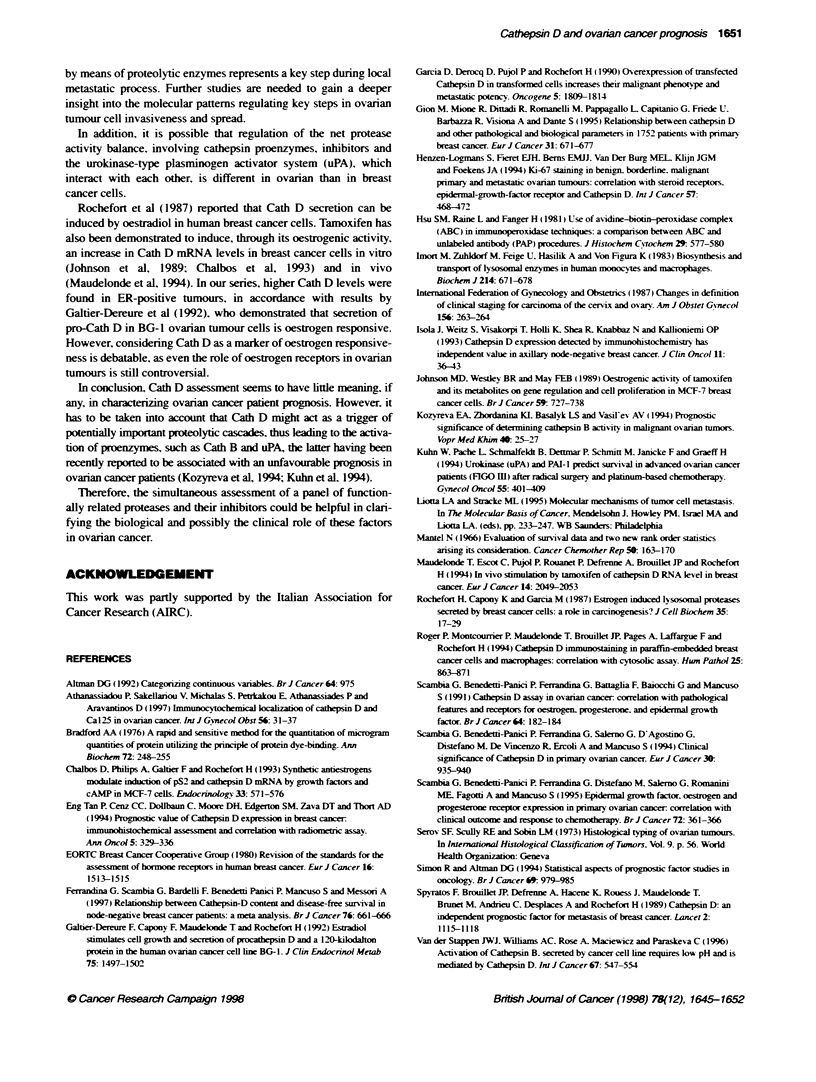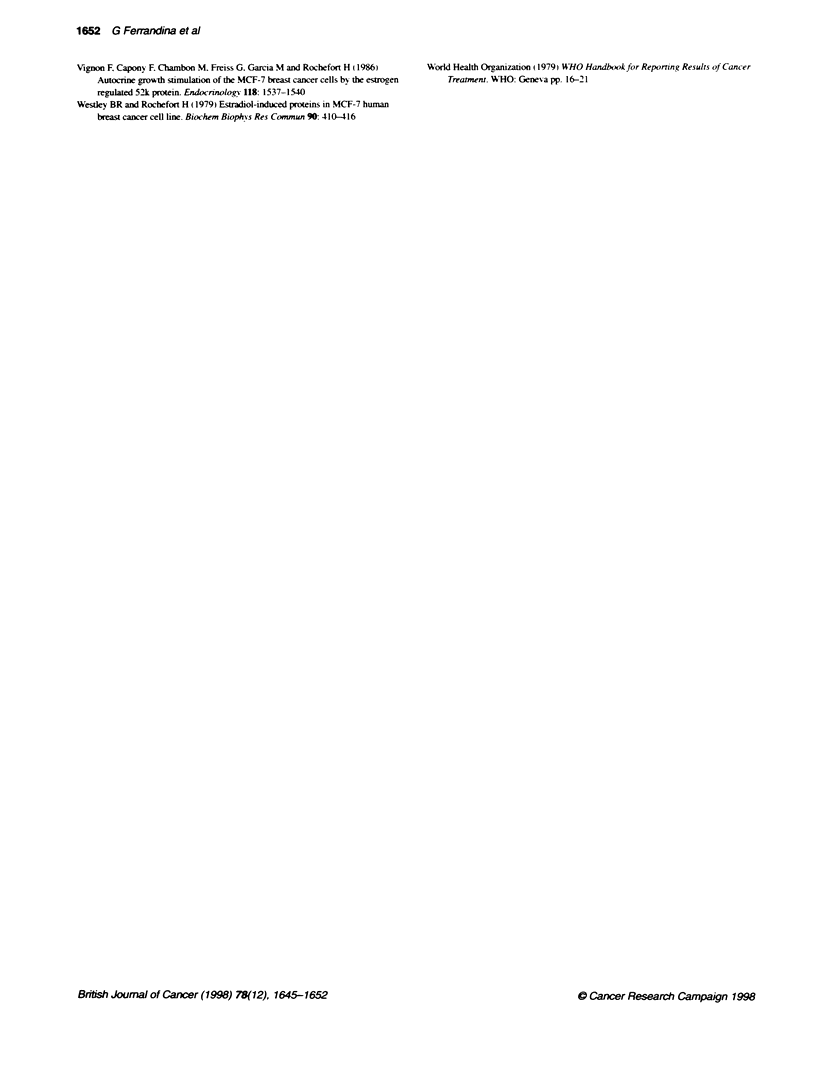# Immunoradiometric and immunohistochemical analysis of Cathepsin D in ovarian cancer: lack of association with clinical outcome.

**DOI:** 10.1038/bjc.1998.737

**Published:** 1998-12

**Authors:** G. Ferrandina, G. Scambia, A. Fagotti, G. D'Agostino, P. Benedetti Panici, A. Carbone, S. Mancuso

**Affiliations:** Department of Gynaecology and Obstetrics, Catholic University, Rome, Italy.

## Abstract

**Images:**


British Journal of Cancer (1998) 78(12), 1645–1652
© 1998 Cancer Research Campaign

### Immunoradiometric and immunohistochemical analysis of Cathepsin D in ovarian cancer: lack of association with clinical outcome

**G Ferrandina[1], G Scambia[1], A Fagotti[1], G D'Agostino[1], P Benedetti Panici[1], A Carbone[2] and S Mancuso[1]**

[1]Department of Gynaecology and Obstetrics, [2]Department of Human Pathology, Catholic University, Rome, Italy

**Summary** The aim of this study was to analyse the clinical significance of Cathepsin D (Cath D) content as determined by an immunoradiometric assay in a series of primary untreated ovarian cancers from 162 patients. In addition, immunohistochemical analysis of Cath D was also performed on a subset of 86 tumours. Cath D levels were distributed in an asymmetrical way and were skewed towards the lower values (median value 20.8 pmol $mg^{-1}$ protein, range 2.0–99.0 pmol $mg^{-1}$ protein). No correlation was found between Cath D levels and clinicopathological parameters. However, the percentage of Cath D positivity was significantly higher in oestrogen receptor-positive (57%) compared with oestrogen receptor-negative (36%) cases ($P = 0.01$). The percentage of Cath D-positive staining was not significantly different for both epithelial (27%) and stromal components (40%). Immunoradiometrically detected Cath D levels were not different according to Cath D stromal immunostaining ($P = 0.18$), while higher Cath D levels were measured in Cath D-positive than in Cath D-negative tumour epithelial cells ($P = 0.027$). Survival analysis was conducted on 161 primary untreated ovarian cancer patients. The 5-year overall survival rate was 57% and 55% in Cath D-positive and Cath D-negative patients respectively ($P = 0.69$). As far as time to progression was concerned, there was no significant difference in the survival rate of patients with either high or low Cath D content ($P = 0.56$). Similar results have been obtained in the subset of patients in which Cath D was analysed by immunohistochemistry. In conclusion, Cath D measurement in tumour extracts appears to have a limited usefulness in improving the prognostic characterization of ovarian cancer patients.

**Keywords:** Cathepsin D; ovarian cancer prognosis

Proteolytic enzymes, which are involved in basement membrane and extracellular matrix degradation, may affect tumour invasiveness and metastatic potential (Liotta et al, 1995). Among the different classes of proteases, particular attention has focused on cathepsins, a family of enzymes that include cystein-proteases, such as Cathepsin B (Cath B), and aspartyl-proteases, such as Cathepsin D (Cath D).

Cath D, first identified as a 52-kDa oestrogen-regulated glycoprotein (Westley et al, 1979), displayed both proteolytic and autocrine mitogenic activity in breast cancer cells in vitro (Vignon et al, 1986). The involvement of Cath D in cancer invasion has also been supported by the demonstration that transfection of tumorigenic rat cells with Cath D cDNA increases their metastatic potential in animal models (Garcia et al, 1990).

The possible clinical significance of Cath D expression was first investigated in breast cancer patients in whom high Cath D levels were reported to be associated with metastatic lymph node involvement, high risk of relapse and poor prognosis (Spyratos et al, 1989; Isola et al, 1993; Gion et al, 1995).

Regarding ovarian cancer, there is some in vitro evidence that oestrogen-stimulated ovarian tumour cells are able to secrete Cath D into the culture medium (Galtier-Dereure et al, 1992), suggesting that cathepsins may play a role in ovarian cancer cell biology. In addition, it has been reported that Cath D is able to activate the precursor form of Cath B (Van der Stappen et al, 1996) and that higher Cath B activity is associated with an increased percentage of relapses in ovarian cancer patients (Kozyreva et al, 1994).

In addition, higher Cath D concentrations have been found in cytosolic extracts from omental metastases than from primary ovarian tumours, as reported by us and other authors (Scambia et al, 1991; Henzen-Logmans et al, 1994). Moreover, the possible association of Cath D with ovarian cancer patient prognosis has been only preliminarily investigated (Scambia et al, 1994).

The aim of this study was to analyse the clinical significance of Cath D content determined by an immunoradiometric assay in a large series of primary untreated ovarian tumours from 162 patients. The correlation between Cath D and biological parameters, such as oestrogen receptor (ER), progesterone receptor (PR) and epidermal growth factor receptor (EGFR), were also investigated.

In addition, the specific contribution of tumour epithelial and adjacent stromal cells to total Cath D content was studied by immunohistochemistry on a subset of 86 cases, and the possible relationship between immunohistochemical and immunoradiometric results was also analysed.

### MATERIALS AND METHODS

One hundred and sixty-two previously untreated patients with histologically confirmed diagnosis of ovarian carcinoma were admitted to the study. Seventy-two cases were from our previous study (Scambia et al, 1994). None of the patients had received chemotherapy previously. All patients were staged according to the FIGO (International Federation of Gynecology and Obstetrics,

*Received 3 June 1997*
*Accepted 24 September 1997*

*Correspondence to:* S Mancuso, Department of Gynaecology and Obstetrics, Catholic University of the Sacred Heart, L.go Gemelli, 8, 00168 Rome, Italy

**Table 1** Distribution of Cathepsin D levels according to clinicopathological characteristics in ovarian cancer

| Variable | Cathepsin D (pmol mg$^{-1}$ cytosolic protein) No. | Mean | Median | Standard deviation | Range | P-value |
|---|---|---|---|---|---|---|
| All cases | 162 | 25.8 | 20.8 | 18.4 | 2.0–99.0 | |
| Histotype | | | | | | |
| Serous | 112 | 24.2 | 17.1 | 17.9 | 2.0–99.0 | |
| Mucinous | 8 | 22.8 | 18.8 | 11.4 | 11.4–41.9 | |
| Endometrioid | 20 | 19.9 | 19.1 | 11.4 | 3.9–35.6 | NS |
| Undifferentiated | 13 | 28.2 | 30.3 | 11.3 | 14.5–46.0 | |
| Other | 9 | 22.9 | 22.9 | 21.3 | 8.0–54.4 | |
| Grading | | | | | | |
| 1–2 | 39 | 24.4 | 20.2 | 14.7 | 4.6–72.9 | NS |
| 3 | 108 | 25.1 | 18.0 | 19.0 | 2.0–99.0 | |
| Not available | 15 | 34.6 | 28.8 | 21.4 | 4.4–84.4 | |
| FIGO stage | | | | | | |
| I–II | 29 | 20.9 | 15.7 | 11.9 | 4.4–54.4 | |
| III | 112 | 24.9 | 19.8 | 17.2 | 2.0–85.0 | 0.026 |
| IV | 21 | 37.4 | 27.5 | 26.8 | 6.1–99.0 | |
| Ascites | | | | | | |
| No | 69 | 23.3 | 21.5 | 12.3 | 2.0–64.0 | NS |
| Yes | 93 | 27.3 | 18.0 | 21.7 | 3.9–99 | |
| Residual tumour[a] | | | | | | |
| ≤ 2 cm | 96 | 25.2 | 20.8 | 17.8 | 2.0–99.0 | NS |
| > 2 cm | 43 | 28.6 | 20.6 | 22.3 | 4.2–99.0 | |
| Response to chemotherapy[a] | | | | | | |
| Complete | 57 | 25.6 | 20.2 | 18.3 | 5.6–99.0 | |
| Partial | 39 | 23.2 | 19.8 | 14.9 | 2.0–54.2 | NS |
| No Change/progression | 34 | 31.2 | 25.0 | 24.4 | 3.9–99.0 | |

[a]Only stage II–IV cases.

1987) classification and histologically classified according to the World Health Organization (WHO) (Serov et al. 1979). In some cases ($n$ = 15), it was impossible to obtain the information relative to histopathological grading from the pathology reports.

All patients underwent maximal cytoreductive effort, and analyses relative to residual tumour were all performed considering only stage II–IV patients ($n$ = 139). Only stage II–IV patients underwent chemotherapy, which was instituted 2–3 weeks after surgery. Gynaecological examination, abdominopelvic ultrasonography, CA 125 assay and radiological investigation, if necessary, were performed monthly for the clinical assessment of response, which was recorded according to WHO criteria (World Health Organization, 1979). Approximately 28 days after the last course, clinically complete responders underwent second-look laparoscopy. In laparoscopy-negative cases, second-look laparotomy was performed for the assessment of pathological response. In nine patients, a second-look laparotomy was not performed because of patient refusal for a second surgery. During laparotomy, after peritoneal washing and a careful inspection of the abdominal cavity, a biopsy of all suspicious lesions was performed and, in the case of no evidence of disease, at least 20 random biopsies were taken. Patients who initially had only an explorative laparotomy underwent a second laparotomy after chemotherapy, and a second cytoreduction was attempted.

### Tumour tissue processing

At the time of surgery, the tumour specimens were dissected and divided into two parts: one part was frozen in liquid nitrogen and stored at – 80°C to be used for receptor detection and the other part was fixed for 24 h in neutral-buffered formalin. After fixation, blocks were routinely paraffin embedded.

### Preparation of cytosol and membrane fractions

Briefly, tumour specimens were finely minced and homogenized in five volumes of ice-cold buffer [25 mM Tris, 1.5 mM EDTA, 5 mM sodium azide, 0.1% monothioglycerol and 20% glycerol (TENMG), pH = 7.4] by applying several intermittent bursts of an Ultra-Turrax homogenizer. The crude homogenate was centrifuged at 7000 $g$ for 20 min at 0°C. The supernatants were then centrifuged at 105 000 $g$ for 75 min at 0°C, obtaining the cytosolic fraction and the membrane pellet (Scambia et al. 1995).

### Immunoradiometric assay

Cath D concentration was assayed in the cytosolic fraction using a solid-phase two-site immunoradiometric assay (CIS Bioindustries, Gift-Sur-Yvette, France). Protein concentration was measured by the Bradford method (Bradford, 1976), using bovine

 © Cancer Research Campaign 1998

**Table 2** Distribution of Cathepsin D positivity according to clinicopathological characteristics in ovarian cancer

| Variable | No. | Cathepsin D positivity: Tumour epithelial cells No. (%) | P-value | Tumour-adjacent stromal cells No. (%) | P-value |
|---|---|---|---|---|---|
| All cases | 86 | 23 (27) | | 35 (40) | |
| Hystotype | | | | | |
| Serous | 61 | 15 (25) | | 22 (37) | |
| Mucinous | 4 | 1 (25) | NS | 2 (50) | NS |
| Endometrioid | 10 | 2 (22) | | 5 (55) | |
| Undifferentiated | 8 | 3 (42) | | 2 (28) | |
| Other | 3 | – | – | | |
| Grading | | | | | |
| 1–2 | 20 | 3 (15) | NS | 6 (30) | NS |
| 3 | 58 | 18 (30) | | 26 (44) | |
| Not available | 8 | – | – | | |
| FIGO stage | | | | | |
| I–II | 18 | 6 (33) | | 9 (44) | |
| III | 54 | 13 (24) | NS | 21 (39) | NS |
| IV | 14 | 4 (28) | | 6 (43) | |
| Ascites | | | | | |
| No | 35 | 9 (26) | NS | 16 (46) | NS |
| Yes | 51 | 14 (27) | | 19 (37) | |
| Residual tumour[a] | | | | | |
| ≤ 2 cm | 54 | 15 (28) | NS | 23 (43) | NS |
| > 2 cm | 17 | 2 (12) | | 8 (47) | |
| Response to chemotherapy[a] | | | | | |
| Complete | 29 | 7 (24) | | 8 (27) | |
| Partial | 19 | 5 (25) | NS | 11 (58) | 0.07 |
| No Change/progression | 19 | 4 (21) | | 10 (53) | |

[a]Only stage II–IV cases.

serum albumin as standard, and was reset to approximately 1 mg ml$^{-1}$ before Cath D assay. Cytosols were then diluted 1:40 and 1:80 with the diluent contained in the kit. Radioactivity was measured in a γ-counter for 1 min. Intra- and interassay variations were 5.8% and 8.9% respectively.

### Receptor assay

ER and PR were measured using the dextran-coated charcoal (DCC) assay according to the European Organization for the Research and Treatment of Cancer (EORTC) protocol (EORTC Breast Cancer Cooperative Group, 1980). EGFR was assayed as previously described (Scambia et al, 1995). Values of 5, 10 and 1.5 fmol mg$^{-1}$ of protein were used to define, respectively, ER, PR and EGFR positivity.

### Immunohistochemical assay

Immunohistochemical staining was performed on formalin-fixed, paraffin-embedded sections from a subset of 86 ovarian cancer patients whose characteristics did not differ from those described in the total population.

The avidin–biotin–peroxidase complex method (ABC) (Hsu et al, 1981) (DAKO, Carpinteria, CA, USA) and the specific polyclonal antihuman cathepsin D antibody (Dako, Carpinteria, USA) were used for immunohistochemistry. The specificity of the immunostaining was controlled with a preadsorption experiment in which anti-Cath polyclonal antibody was preincubated with a 100-fold molar excess of purified Cathepsin D. Immunostaining of tumour cells as well as macrophages was abolished.

Five-micrometre sections were dewaxed in xylene, rehydrated in descending concentration of alcohol down to 80%, washed in tap and distilled water, and treated with 0.3% hydrogen peroxide in methanol for 25 min to remove endogenous peroxidase activity. The sections were then washed in Tris-buffered saline (TBS) (pH 7.6) and incubated with normal serum as the blocking reagent to minimize non-specific binding. A 1:200 dilution of the specific polyclonal rabbit anti-human Cath D antibody was applied for 30 min at room temperature. The sections were then incubated with the biotinylated goat anti-rabbit IgG and with avidin–biotin–peroxidase complex for 10 min at room temperature. Finally, the sections were washed in TBS, stained by incubation with diaminobenzidine for 10 min and then counterstained with haematoxylin. Normal rabbit immunoglobulin G (IgG; Sigma, St Louis, MO, USA) was used as a negative control.

### Cath D immunostaining evaluation

Separate evaluation of neoplastic and stromal (i.e. fibroblasts and mononuclear cells) elements was performed by means of light-microscopic examination.

The sections were examined independently by two observers (AC and AF) and scored according to the intensity of staining and proportion of cells stained. In case of disagreement, the sections

© Cancer Research Campaign 1998

**Figure 1** Distribution of Cath D levels in 162 primary ovarian tumours. The class interval is 10 pmol mg$^{-1}$ protein were reviewed on a two-headed microscope (Laborlux-S, Leitz) to reach a consensus. In detail, the cases were scored as negative when no staining was observed. When reactivity was observed in less than 25% of tumour cells or in few stromal cells, the cases were scored 1+. When the positivity was observed in 25–75% of tumour cells or in a moderate number of stromal cells, the cases were scored 2+. When > 75% of tumour cells or many stromal cells were positive, the cases were scored 3+.

In this study, cases with scores between – and 1+ were considered as negative, while cases with scores between 2+ and 3+ were considered as Cath D positive. The interpretation of the immunostaining was determined without any knowledge of the clinicopathological and biochemical parameters or of the follow-up data.

### Statistical analysis

Kruskal–Wallis and Mann–Whitney non-parametric tests were used to analyse the distribution of cytosolic Cath D content according to clinicopathological characteristics of the patients. Mann–Whitney non-parametric tests was used to analyse the distribution of ER, PR and EGFR, according to Cath D status. The Fisher exact test for proportion was used to analyse the distribution of Cath D-positive cases in epithelial and in stromal cells in relation to clinicopathological characteristics and receptor status. All medians and life-tables were calculated using the product-limit estimate, and the curves were examined by means of the log-rank test (Mantel, 1966). Cox's analysis was used to evaluate the prognostic significance of Cath D as a continuous variable. Time to progression and overall survival were calculated from the day of the first surgery to the date of clinical or pathological progression or death. All survival analyses were performed using Solo Statistical Software (BMDP Statistical Software, Los Angeles, CA, USA).

### RESULTS

### Immunoradiometric analysis

The distribution of Cath D levels in 162 primary ovarian tumours, including previously described tumours (Scambia et al, 1994), is shown in Figure 1. Cath D levels were distributed in an asymmetrical way and were skewed towards the lower values (median value 20.8 pmol mg$^{-1}$ protein, range 2.0–99.0 pmol mg$^{-1}$ protein).

**Table 3** Distribution of immunoradiometrically detected Cathepsin D levels according to immunohistochemical Cathepsin D status

| Tumour cells | Cathepsin D (pmol mg$^{-1}$ cytosolic protein) No. | Median | Range | $P$-value |
|---|---|---|---|---|
| Epithelial | | | | |
| Positive | 23 | 32.8 | 10.7–72.6 | 0.027 |
| Negative | 63 | 20.8 | 3.9–99.0 | |
| Stromal | | | | |
| Positive | 35 | 22.9 | 3.9–99.0 | 0.18 |
| Negative | 51 | 24.7 | 4.4–99.0 | |

The association between Cath D levels and recognized prognostic factors is shown in Table 1. No correlation was found with grading, histology, presence of ascites and residual tumour. In addition, the lack of association between Cath D and these clinicopathological characteristics was confirmed by referring to Cath D positivity as defined by using different cut-off values.

Cath D levels were significantly higher in stage III (median 19.8, range 2.0–85.0 pmol mg$^{-1}$ cytosolic protein) and stage IV (median 27.5, range 6.1–99.0 pmol mg$^{-1}$ cytosolic protein) patients compared with stage I–II (median 15.7, range 4.4–54.4 pmol mg$^{-1}$ cytosolic protein) cases ($P = 0.026$).

We did not observe any correlation between Cath D values and response to chemotherapy, as reported in Table 1.

The association between Cath D content and steroid hormone and EGF receptors measured in the cytosolic fraction was also investigated; the percentage of Cath D positivity was significantly higher in ER-positive (57%) than in ER-negative (36%) cases ($P = 0.01$). Also, when PR and EGFR levels were assayed, no correlation was found to exist with Cath D levels.

### Immunohistochemical analysis

Figure 2 shows a representative example of a Cath D immunohistochemical staining in a primary ovarian cancer. A specific cytoplasmic staining was observed in epithelial as well as in stromal cells. Cath D-positive stromal cells were mainly represented by reactive fibrohistiocytic cells and macrophages that were concentrated immediately adjacent to tumour cells.

The percentage of Cath D-positive epithelium was 27% (23 out of 86 patients), and this was calculated not to be significantly different with respect to the percentage of Cath D positivity in stromal cells (35 out of 86 patients) (40%). In addition, there was no association between Cath D epithelial and Cath D stromal staining (data not shown).

When Cath D staining in the epithelium was evaluated according to clinicopathological parameters, no relationship appeared to exist between the presence or absence of Cath D staining and any clinical parameters (Table 2). However, patients whose tumours showed positively stained stroma demonstrated a trend towards partial or no response to chemotherapy ($P = 0.07$).

Steroid hormone receptor and EGFR levels were not differently distributed according to Cath D status in epithelial and stromal cells (data not shown).

© Cancer Research Campaign 1998

**Figure 2** Immunohistochemical staining of Cath D in primary human ovarian cancer (ABC method). (**A** and **C**) Positive reaction for Cath D is seen predominantly in epithelial tumour cells (original magnification **A**, ×400; **C** ×100). (**B** and **D**) Positive reaction for Cath D is seen predominantly in intratumoral histiocytes and fibroblasts (original magnification **B**, ×400; **D**, ×100)

### Relationship between immunoradiometric and immunohistochemical assay

A relationship was observed between Cath D levels measured by immunoradiometry and epithelial Cath D content detected by immunohistochemistry ($P = 0.027$), while this relationship did not exist for stromal cells ($P = 0.18$) (Table 3).

### Survival analysis

Survival analysis was conducted on 161 primary untreated ovarian cancer patients. One patient died from intercurrent disease. During follow-up, death and progression of disease was observed in 55 and 85 patients respectively.

Different cut-off values, corresponding to the upper, the lower quartiles and the median Cath D levels were tested to distinguish low vs high Cath D content groups.

Figure 3A shows the overall survival curves in relation to Cath D cytosolic status, defined using a cut-off of 21 pmol $mg^{-1}$ protein, corresponding to the median value. The 5-year overall survival was 57% and 55% in Cath D-positive and Cath D-negative patients respectively ($P = 0.69$). Similar results were obtained at any cut-off tested (data not shown).

As far as time to progression is concerned, no significant difference in the survival rate of patients with high or low Cath D content was observed, as shown in Figure 3B ($P = 0.56$). Cox's Hazard regression analysing Cath D as a continuous variable gave similar results (data not shown).

Survival analysis was also conducted on the subgroup of 86 patients in whom Cath D was analysed by immunohistochemistry. The 5-year time-to-progression rate was 23% for epithelial Cath D-positive compared with 38% for epithelial Cath D-negative cases ($P = 0.91$) and 30% for stromal Cath D-positive compared with 37% for stromal Cath D-negative cases ($P = 0.67$). The 5-year overall survival was not significantly different between Cath D-positive and Cath D-negative cases for both epithelial ($P = 0.94$) and stromal ($P = 0.67$) components.

*© Cancer Research Campaign 1998*

**Figure 3** Overall survival (**A**) and time-to-progression (**B**) according to cytosolic Cath D status in primary ovarian cancer patients

### DISCUSSION

To our knowledge, this is the first study to investigate the possible clinical role of Cath D content in a large series of primary untreated ovarian cancer patients by means of both immunoradiometric and immunohistochemical assays.

The present study, which also includes previously analysed cases (Scambia et al, 1994), was conceived to clarify the possible association between Cath D and ovarian cancer patient clinical outcome, as our preliminary findings only demonstrated a borderline and a not easily explainable association between cytosolic Cath D levels and prognosis in ovarian cancer.

In the present report, which refers to a larger sample population observed for a longer follow-up period, the potential clinical significance of Cath D expression has been analysed using several Cath D cut-off points and, more importantly, by using Cath D as a continuous variable. The latter approach has the advantage of retaining all the information of the biological markers and excludes the bias due to preselection of the best discriminating cut-off points (Altman et al, 1992). Data reported here suggest that the assessment of total cytosolic Cath D levels in tumour extracts has limited usefulness in improving the prognostic characterization of ovarian cancer patients, thus emphasizing the need to be careful when reporting the prognostic value of a new biological factor based on a pilot study. Large, multi-institutional, confirmatory (phase III) studies have been recommended to reach reliable conclusions about the possible clinical usefulness of biological factors as new prognostic indicators in human malignancies (Simon et al, 1994).

Our results have been obtained by means of a biochemical assay, measuring Cath D levels in total cytosolic fractions from tumour samples. Therefore, it is possible that the tumour samples contain invading stromal and inflammatory cell contaminants, which have been reported to express high levels of Cath D (Imort et al, 1983), possibly leading to an inappropriate estimation of the role of this protease.

Only scanty data have been reported until now on the immunohistochemical evaluation of Cath D content in ovarian cancer (Athanassiadou et al, 1997) and the relative contribution of epithelial and stromal cells to total Cath D expression levels. Also the correlation between cytosolic Cath D levels of a tumour and specific Cath D immunostaining have not been analysed.

In order to investigate whether epithelial and stromal Cath D content can be independently associated to ovarian cancer patient clinical outcome, we analysed Cath D expression by means of immunohistochemistry on a subset of primary ovarian tumours that had also been evaluated for their cytosolic Cath D content.

We demonstrated that, in our series, cytosolic Cath D levels were directly associated with immunohistochemically detected Cath D content for the epithelial tumour cells, as reported by several authors for breast cancer (Eng Tan et al, 1994; Roger et al, 1994). On the other hand, this association has not been found for stromal cells, thus indicating that epithelial cell Cath D content mainly contributes to total Cath D in tumour extracts.

Overall, using both methods of determining Cath D content in ovarian tumours, no correlation was found to exist between Cath D levels and patient outcome.

Our data are different to those reported for breast cancer patients in whom, in spite of some discrepancies, there has been general agreement to consider high Cath D expression as being a marker of unfavourable clinical outcome (Ferrandina et al, 1997).

Patterns of ovarian cancer spread are rather peculiar as ovarian tumour cells thrive in the abdominal cavity microenvironment and can easily reach distant sites on peritoneal surfaces. Moreover, the biological and clinical role of lymphatic dissemination in ovarian cancer natural history is yet to be fully clarified. In this context, the different clinical significance of Cath D expression in breast and ovarian tumours could be a result of the different biological roles that the protease system might play in these two neoplasias. It is conceivable that the degradative enzyme machinery may not be as relevant for ovarian cancer cells as for breast cancer cells, in which the degradation of basement membrane and extracellular matrix

© Cancer Research Campaign 1998

by means of proteolytic enzymes represents a key step during local metastatic process. Further studies are needed to gain a deeper insight into the molecular patterns regulating key steps in ovarian tumour cell invasiveness and spread.

In addition, it is possible that regulation of the net protease activity balance, involving cathepsin proenzymes, inhibitors and the urokinase-type plasminogen activator system (uPA), which interact with each other, is different in ovarian than in breast cancer cells.

Rochefort et al (1987) reported that Cath D secretion can be induced by oestradiol in human breast cancer cells. Tamoxifen has also been demonstrated to induce, through its oestrogenic activity, an increase in Cath D mRNA levels in breast cancer cells in vitro (Johnson et al, 1989; Chalbos et al, 1993) and in vivo (Maudelonde et al, 1994). In our series, higher Cath D levels were found in ER-positive tumours, in accordance with results by Galtier-Dereure et al (1992), who demonstrated that secretion of pro-Cath D in BG-1 ovarian tumour cells is oestrogen responsive. However, considering Cath D as a marker of oestrogen responsiveness is debatable, as even the role of oestrogen receptors in ovarian tumours is still controversial.

In conclusion, Cath D assessment seems to have little meaning, if any, in characterizing ovarian cancer patient prognosis. However, it has to be taken into account that Cath D might act as a trigger of potentially important proteolytic cascades, thus leading to the activation of proenzymes, such as Cath B and uPA, the latter having been recently reported to be associated with an unfavourable prognosis in ovarian cancer patients (Kozyreva et al, 1994; Kuhn et al, 1994).

Therefore, the simultaneous assessment of a panel of functionally related proteases and their inhibitors could be helpful in clarifying the biological and possibly the clinical role of these factors in ovarian cancer.

### ACKNOWLEDGEMENT

This work was partly supported by the Italian Association for Cancer Research (AIRC).

### REFERENCES

Altman DG (1992) Categorizing continuous variables. *Br J Cancer* **64**: 975

Athanassiadou P, Sakellariou V, Michalas S, Petrkakou E, Athanassiades P and Aravantinos D (1997) Immunocytochemical localization of cathepsin D and Ca125 in ovarian cancer. *Int J Gynecol Obst* **56**: 31–37

Bradford AA (1976) A rapid and sensitive method for the quantitation of microgram quantities of protein utilizing the principle of protein dye-binding. *Ann Biochem* **72**: 248–255

Chalbos D, Philips A, Galtier F and Rochefort H (1993) Synthetic antiestrogens modulate induction of pS2 and cathepsin D mRNA by growth factors and cAMP in MCF-7 cells. *Endocrinology* **33**: 571–576

Eng Tan P, Cenz CC, Dollbaun C, Moore DH, Edgerton SM, Zava DT and Thort AD (1994) Prognostic value of Cathepsin D expression in breast cancer: immunohistochemical assessment and correlation with radiometric assay. *Ann Oncol* **5**: 329–336

EORTC Breast Cancer Cooperative Group (1980) Revision of the standards for the assessment of hormone receptors in human breast cancer. *Eur J Cancer* **16**: 1513–1515

Ferrandina G, Scambia G, Bardelli F, Benedetti Panici P, Mancuso S and Messori A (1997) Relationship between Cathepsin-D content and disease-free survival in node-negative breast cancer patients: a meta analysis. *Br J Cancer* **76**: 661–666

Galtier-Dereure F, Capony F, Maudelonde T and Rochefort H (1992) Estradiol stimulates cell growth and secretion of procathepsin D and a 120-kilodalton protein in the human ovarian cancer cell line BG-1. *J Clin Endocrinol Metab* **75**: 1497–1502

Garcia D, Derocq D, Pujol P and Rochefort H (1990) Overexpression of transfected Cathepsin D in transformed cells increases their malignant phenotype and metastatic potency. *Oncogene* **5**: 1809–1814

Gion M, Mione R, Dittadi R, Romanelli M, Pappagallo L, Capitanio G, Friede U, Barbazza R, Visiona A and Dante S (1995) Relationship between cathepsin D and other pathological and biological parameters in 1752 patients with primary breast cancer. *Eur J Cancer* **31**: 671–677

Henzen-Logmans S, Fieret EJH, Berns EMJJ, Van Der Burg MEL, Klijn JGM and Foekens JA (1994) Ki-67 staining in benign, borderline, malignant primary and metastatic ovarian tumours: correlation with steroid receptors, epidermal-growth-factor receptor and Cathepsin D. *Int J Cancer* **57**: 468–472

Hsu SM, Raine L and Fanger H (1981) Use of avidine–biotin–peroxidase complex (ABC) in immunoperoxidase techniques: a comparison between ABC and unlabeled antibody (PAP) procedures. *J Histochem Cytochem* **29**: 577–580

Imort M, Zuhldorf M, Feige U, Hasilik A and Von Figura K (1983) Biosynthesis and transport of lysosomal enzymes in human monocytes and macrophages. *Biochem J* **214**: 671–678

International Federation of Gynecology and Obstetrics (1987) Changes in definition of clinical staging for carcinoma of the cervix and ovary. *Am J Obstet Gynecol* **156**: 263–264

Isola J, Weitz S, Visakorpi T, Holli K, Shea R, Knabbaz N and Kallioniemi OP (1993) Cathepsin D expression detected by immunohistochemistry has independent value in axillary node-negative breast cancer. *J Clin Oncol* **11**: 36–43

Johnson MD, Westley BR and May FEB (1989) Oestrogenic activity of tamoxifen and its metabolites on gene regulation and cell proliferation in MCF-7 breast cancer cells. *Br J Cancer* **59**: 727–738

Kozyreva EA, Zhordanina KI, Basalyk LS and Vasil'ev AV (1994) Prognostic significance of determining cathepsin B activity in malignant ovarian tumors. *Vopr Med Khim* **40**: 25–27

Kuhn W, Pache L, Schmalfeldt B, Dettmar P, Schmitt M, Janicke F and Graeff H (1994) Urokinase (uPA) and PAI-1 predict survival in advanced ovarian cancer patients (FIGO III) after radical surgery and platinum-based chemotherapy. *Gynecol Oncol* **55**: 401–409

Liotta LA and Stracke ML (1995) Molecular mechanisms of tumor cell metastasis. In *The Molecular Basis of Cancer*, Mendelsohn J, Howley PM, Israel MA and Liotta LA. (eds), pp. 233–247. WB Saunders: Philadelphia

Mantel N (1966) Evaluation of survival data and two new rank order statistics arising its consideration. *Cancer Chemother Rep* **50**: 163–170

Maudelonde T, Escot C, Pujol P, Rouanet P, Defrenne A, Brouillet JP and Rochefort H (1994) In vivo stimulation by tamoxifen of cathepsin D RNA level in breast cancer. *Eur J Cancer* **14**: 2049–2053

Rochefort H, Capony K and Garcia M (1987) Estrogen induced lysosomal proteases secreted by breast cancer cells: a role in carcinogenesis? *J Cell Biochem* **35**: 17–29

Roger P, Montcourrier P, Maudelonde T, Brouillet JP, Pages A, Laffargue F and Rochefort H (1994) Cathepsin D immunostaining in paraffin-embedded breast cancer cells and macrophages: correlation with cytosolic assay. *Hum Pathol* **25**: 863–871

Scambia G, Benedetti-Panici P, Ferrandina G, Battaglia F, Baiocchi G and Mancuso S (1991) Cathepsin D assay in ovarian cancer: correlation with pathological features and receptors for oestrogen, progesterone, and epidermal growth factor. *Br J Cancer* **64**: 182–184

Scambia G, Benedetti-Panici P, Ferrandina G, Salerno G, D'Agostino G, Distefano M, De Vincenzo R, Ercoli A and Mancuso S (1994) Clinical significance of Cathepsin D in primary ovarian cancer. *Eur J Cancer* **30**: 935–940

Scambia G, Benedetti-Panici P, Ferrandina G, Distefano M, Salerno G, Romanini ME, Fagotti A and Mancuso S (1995) Epidermal growth factor, oestrogen and progesterone receptor expression in primary ovarian cancer: correlation with clinical outcome and response to chemotherapy. *Br J Cancer* **72**: 361–366

Serov SF, Scully RE and Sobin LM (1973) Histological typing of ovarian tumours. In *International Histological Classification of Tumors*, Vol. 9, p. 56. World Health Organization: Geneva

Simon R and Altman DG (1994) Statistical aspects of prognostic factor studies in oncology. *Br J Cancer* **69**: 979–985

Spyratos F, Brouillet JP, Defrenne A, Hacene K, Rouess J, Maudelonde T, Brunet M, Andrieu C, Desplaces A and Rochefort H (1989) Cathepsin D: an independent prognostic factor for metastasis of breast cancer. *Lancet* **2**: 1115–1118

Van der Stappen JWJ, Williams AC, Rose A, Maciewicz and Paraskeva C (1996) Activation of Cathepsin B, secreted by cancer cell line requires low pH and is mediated by Cathepsin D. *Int J Cancer* **67**: 547–554

Vignon F, Capony F, Chambon M, Freiss G, Garcia M and Rochefort H (1986) Autocrine growth stimulation of the MCF-7 breast cancer cells by the estrogen regulated 52k protein. *Endocrinology* **118**: 1537–1540

Westley BR and Rochefort H (1979) Estradiol-induced proteins in MCF-7 human breast cancer cell line. *Biochem Biophys Res Commun* **90**: 410–416

World Health Organization (1979) *WHO Handbook for Reporting Results of Cancer Treatment*. WHO: Geneva pp. 16–21

© Cancer Research Campaign 1998